# Evaluating the association of vitamin D3, parathyroid hormone, and C-reactive protein serum levels in patients with an acute psychotic episode: a cross-sectional study in tertiary centre in Iran

**DOI:** 10.1186/s12888-023-05234-5

**Published:** 2023-10-06

**Authors:** Shahrzad Arya, Amirhossein Kamyab, Seyed Amir Sanatkar, Mohammad Pourmehdiardebili, Alireza Ebrahimi, Parnia Kamyab, Kaveh Alavi, Zhina Zarei, Hamid Reza Ahmadkhaniha

**Affiliations:** 1 Research Center for Addiction and Risky Behaviors, Iran University of Medical Sciences, Tehran, Iran; 2 Faculty of Medicine, Fasa University of Medical Sciences, Fasa, Iran; 3 Arak University of Medical Sciences, Arak, Iran; 4 Golestan University of Medical Sciences, Gorgan, Iran; 5 Student Research Committee, Shiraz University of Medical Sciences, Shiraz, Iran; 6 Department of Psychiatry, Mental Health Research Center, School of Behavioral Sciences & Mental Health (Tehran Institute of Psychiatry), Iran University of Medical Sciences, Tehran, Iran

**Keywords:** Vitamin D, CRP, Psychiatric disorders, PTH, BPRS, Schizophrenia, Bipolar disorder, With methamphetamine-induced psychotic disorder, Psychosis, Acute psychotic episode

## Abstract

**Background:**

The high impact of vitamin D on brain development and its relationship with inflammatory markers in the clinical course of psychiatric disorders have compelled researchers to investigate the potential association between vitamin D levels, C-reactive protein (CRP) levels, and the incidence of mental disorders. In the present study, we aimed to compare the serum levels of vitamin D and its related markers, including calcium, phosphorus, and parathyroid hormone (PTH), along with CRP, in 3 groups of patients with acute psychotic episodes, including schizophrenia, bipolar disorder, and methamphetamine-induced psychosis, with a standard control group of the Iranian population.

**Methods:**

This descriptive cross-sectional study was conducted at a psychiatric hospital in Tehran, Iran, and involved a total of 185 subjects. The subjects included four groups: acute phase of schizophrenia (n = 49), acute manic episodes of bipolar disorder (n = 43), methamphetamine-induced psychotic disorder (n = 46), and control group (n = 47). Among 138 patients in acute psychotic episodes, 33 patients were in their first episode of psychosis, while 105 patients were in acute exacerbation of their chronic psychotic disorders. The Brief Psychiatric Rating Scale (BPRS) was measured by an expert attending psychiatrist for all patients. Then, serum levels of calcium, phosphorus, parathormone, vitamin D, and CRP were assessed in all study groups.

**Results:**

Among our 185 study subjects, it was observed that individuals with higher education levels and those who were married had a lower prevalence of mental disorders. In all patient groups, the serum levels of CRP were significantly higher, and PTH levels were significantly lower than in the control group (p < 0.001). The serum levels of calcium, phosphorus, and vitamin D were not statistically significantly different between the patient and control groups of the study. In chronic psychotic patients, CRP levels were significantly higher (p < 0.031), and vitamin D levels were significantly lower (p < 0.044) compared to first-episode psychotic patients.

**Conclusion:**

This study suggests that CRP levels are significantly higher and PHT level is significantly lower in acute psychotic patients. Moreover, vitamin D levels were significantly lower in chronic psychotic patients compared to first-episode psychotic patients.

## Background

Mental health disorders are substantial global disease burdens that have wide-ranging impacts on individuals, families, and society at large [1–3]. The associated burden on the healthcare system and the economy is substantial, stemming from the costs of treatment as well as the negative consequences of impaired performance and reduced productivity [4]. Notably, A comprehensive analysis revealed a significant increase in the disability-adjusted life years (DALYs) attributed to mental health disorders globally, rising from 80.8 million to 125.3 million over the past 30 years [5]. The outbreak of COVID-19 has further highlighted the importance of mental health, as the incidence rate of psychiatric disorders has escalated due to the social and economic impacts of the pandemic [6]. Consequently, researchers have been driven to explore both extrinsic and intrinsic factors contributing to the development of mental disorders [7]. Current knowledge underscores the significant roles of genetic factors, biological changes, and environmental stresses in the development of psychological disorders [8, 9].

Vitamin D and its metabolites (25-hydroxyvitamin D3, 1.25-dihydroxyvitamin D3, and 24.25-dihydroxyvitamin D3) have been shown to have a crucial role in maintaining the health of the nervous system as they can cross the blood-brain barrier and have been shown to have a crucial role in maintaining the health of the nervous system [10]. Vitamin D has a regulatory role in the production and release of neurotrophins, the synthesis of neuromediators, intracellular calcium homeostasis, and the prevention of oxidative stress on nerve tissues [11, 12]. Notably, vitamin D deficiency has been found to be more prevalent in patients with bipolar disorder and schizophrenia [13, 14]. with a study suggesting that maternal vitamin D deficiency may be a potential risk factor for schizophrenia in adults [15]. Furthermore, neuroimaging studies have revealed a significant association between vitamin D levels and peripheral gray matter volume in patients with psychosis [16].

The relative association between a sufficient vitamin D level and mental health might be due to its regulatory and anti-inflammatory effects observed in different body systems [17]. Prior observations suggest that psychiatric disorders are associated with inflammatory and autoimmune responses caused by the release of cytokines that could lead to microvascular damage in the brain [18, 19]. The serum levels of Interleukin 1 beta (IL-1β), Interleukin 6 (IL-6), and transforming growth factor beta (TGF-β) were found to be elevated in acute phases of schizophrenia [20]. Furthermore, increased serum levels of Interleukin 4 (IL-4) were detected in patients in the manic phase of bipolar disorder [21]. C-reactive protein (CRP), an acute-phase reactive protein produced by the liver in response to acute inflammation, is the primary indicator of an acute inflammatory process in the body [22]. Several studies have shown a significant association between C-reactive protein (CRP) levels and the development of mental disorders, suggesting this marker can be used as a screening tool for the progression of the disease [23–26]. Previous investigations have also shown that the progression of psychiatric diseases such as bipolar disorder and schizophrenia is caused by inflammatory pathways resulting in microvascular brain damage [20, 27–29]. Given the antioxidative properties of vitamin D and its role in the regulation of intracellular calcium homeostasis and the release of neuromodulators suggest a potential association between this vitamin and the progression of psychiatric disorders [30].

Previous investigations were mainly focused on the association of vitamin D levels and CRP levels, as an inflammatory marker, with schizophrenia and bipolar disorder. However, to the best of our knowledge, limited studies have been conducted to evaluate vitamin D and its related markers, such as calcium, phosphorus, and parathyroid hormone (PTH), as well as inflammatory markers to better understand the pathophysiology and the association of them in acute psychotic patients. Additionally, through a review of the existing literature, a few studies have compared vitamin D, its related markers, and CRP serum levels between patients with newly diagnosed psychotic conditions and those in the acute exacerbation of their chronic psychosis. Furthermore, there is a lack of studies that have specifically examined vitamin D levels in patients with methamphetamine-induced psychotic disorder, and limited research has been conducted on inflammatory markers in this particular population.

Therefore, in this study, we compared serum levels of CRP, vitamin D, and its related markers in patients with various forms of acute psychosis (schizophrenia, bipolar disorder, and methamphetamine-induced psychosis) with a healthy control group. Furthermore, we conducted a comparison of these markers within the patient groups to explore their potential relationship with the onset of mental disorders. In addition, we aimed to investigate the long-term effects of psychosis on these markers by comparing the serum levels of CRP, vitamin D, and its related markers in first-episode psychotic and chronic psychotic patients.

## Methods

### Study Design and participants

This descriptive cross-sectional study was conducted on 138 patients admitted to Iran Psychiatric Hospital, Tehran, Iran. The study groups comprised 49 patients with schizophrenia, 43 patients with bipolar disorder, 46 patients with substance-induced psychotic disorders, and 47 participants from hospital staff and patients’ companions as the control group. The patient groups were experiencing acute psychotic episodes. Among 138 patients in acute psychotic episodes, 33 patients were in their first episode of psychosis, while 105 patients were in acute exacerbation of their chronic psychotic disorders. The patients were interviewed by an expert attending psychiatrist, following the guidelines of the Structured Clinical Interview for Diagnostic and Statistical Manual of Mental Disorders, Fourth Edition (DSM-IV), known as SCID-IV, and were diagnosed with the mentioned psychiatric disorders. The control group was chosen randomly from the mentally healthy hospital staff and patients’ companions following interviews with an expert attending psychiatrist. The companions were not first-degree relatives of the patients. All the participants in both the patient and control groups were selected using the simple random sampling method.

### Inclusion and exclusion criteria

Inclusion criteria were confirmation of clinical diagnosis based on SCID-IV in each group and absence of any of these three categories of disorders in the control group, age between 20 and 30 years, and a disease duration of fewer than 5 years (to reduce the impact of chronicity on CRP, and vitamin D levels); and willingness to participate in the study. The exclusion criteria were consumption of any medication, supplement, use of any recreational drug of abuse, or suffering from any acute or chronic condition that could affect the blood vitamin D level, calcium, phosphorus, PTH, and CRP levels.

### Ethics approval and consent to participate

The participants or their legal guardians were educated about the purpose of the study, and written informed consent was gathered from them. Ethical considerations were approved by the Iran University of Medical Sciences Ethics Committee following the Declaration of Helsinki, with the ethical code: IR.IUMS.1395.1952.

### Data Collection Methods

A questionnaire was designed to gather demographic data, including age, sex, level of education, marital status, smoking history, a brief history of medical diseases or psychiatric disorders, the amount of daily activity, vitamin D-rich dietary intake, and previous medication history.

The mental status of patients was measured by the Brief Psychiatric Rating Scale (BPRS), a reliable tool for assessing the psychopathology of mental disorders [31]. This scale measures the severity of psychiatric symptoms in psychotic patients, considering a wide range of symptoms such as confusion of thoughts, social or emotional isolation, anxiety, hostility, and skepticism. This scale has 18 items, each scoring on a 7-point Likert scale from 0 to 6, and the total score is calculated to be between 0 and 108. The questionnaires were filled out by an expert attending psychiatrist at the hospital.

### Procedure

After obtaining informed consent from the participants, the related questionnaires were completed, and 5 ml of venous blood was taken. The samples were sent to the reference laboratory (Iran Psychiatric Hospital Laboratory, Tehran, Iran) to measure the blood levels of 1.25-dihydroxyvitamin D3, calcium, phosphorus, PTH, and CRP. We considered 1.25-dihydroxyvitamin D3 blood levels less than 10 ng/dl equivalent to deficiency, 10 ng/dl and 20 ng/ml as insufficiency, and 20 ng/ml to 100 ng/ml as normal.

### Data Analysis

The data was analyzed by an expert statistician blinded to the study experiments. T-test or One-Way ANOVA was used to compare quantitative variables. The Chi-square test was also used to compare qualitative variables such as age, marital status, and smoking history, and p-values less than 0.05 were considered to be significant. The analysis was performed using Statistical Package for the Social Sciences (SPSS) version 16.0 software.

## Results

Table 1 summarizes the demographic information of the participants. All participants were between 20 and 30 years old (25.86 ± 2.92, mean ± standard deviation). A statistically significant difference in gender distribution was observed where the majority of patients with methamphetamine-induced psychotic disorder were male (91.3%). Additionally, the distribution of educational levels among the participants revealed that the patients had lower educational levels compared to the control group (p < 0.001).

There was a statistically significant difference in the frequency distribution of marital status among the four investigated groups, where single participants were more predisposed to develop psychotic disorders (p < 0.001). Although most of the participants in the control group were non-smokers, most patients with a methamphetamine-induced psychotic disorder were heavy smokers (p < 0.001). The presence of severe psychiatric patients in first-degree family members in schizophrenic patients (24.5%), bipolar disorder patients (30.2%), methamphetamine-induced psychotic patients (17.4%), and the control group (2.1%) was observed with significantly different ratios (p = 0.004).

**Table 1:** Characteristics of the participants in the study

Variable		Schizophrenia (49)	Bipolar disorder type 1 (43)	Methamphetamine-induced psychotic disorder (46)	Control group (47)
Age (Mean ± SD)		24.92 ± 2.85	25.51 ± 3.44	26.78 ± 2.44	26.28 ± 2.62
Gender	Male	39 (79.6%)	28 (65.1%)	42 (91.3%)	30 (63.8%)
	Female	10 (20.4%)	15 (34.9%)	4 (8.7%)	17 (36.2%)
Educational level	Primary education	10 (20.4%)	5 (11.6%)	10 (21.7%)	0
	High school education	34 (69.4%)	26 (60.5%)	30 (65.2%)	15 (31.9%)
	University education	5(10.2%)	12(27.9%)	6(13.0%)	32(68.1%)
Marital status	Single	48 (98.0%)	37 (86.0%)	39 (84.8%)	28 (59.6%)
	Married	1 (2.0%)	6 (14.0%)	7 (15.2%)	19 (40.4%)
Smoking	Non-smokers	24 (49.0%)	25 (58.1%)	5 (10.9%)	40 (85.1%)
	Light smokers (1 to 9 pack years)	23 (46.9%)	11 (25.6%)	21 (45.7%)	7 (14.9%)
	Heavy smokers (10 to 20 pack years)	2* (1.4%)	7 (16.3%)	20 (43.5%)	0
Frequency of hospitalization	One time	19 (38.8%)	21 (48.8%)	26 (56.5%)	
	2–3 times	24 (40.9%)	17 (39.5%)	17 (37.0%)	
	More than 3 times	6 (12.2%)	5 (11.6%)	3 (6.5%)	
Duration of illness	Less than one year	3 (6.1%)	12 (27.9%)	10 (21.7%)	
	1–3 years	24 (49.0%)	12 (27.9%)	18 (39.1%)	
	More than 3 years	22 (44.9%)	19 (44.2%)	18 (39.1%)	
Positive family history of severe psychotic disorders		12 (24.5%)	13 (30.2%)	8 (17.4%)	1 (2.1%)

Vitamin D levels in the four study groups are shown in Table 2. No significant difference was observed in vitamin D levels between the four groups of the study and the subgroups of men and women. Furthermore, vitamin D levels were not significantly correlated with age, disease severity based on BPRS, duration of illness, or the number of hospitalizations.

**Table 2:** Vitamin D levels in the participants

Vitamin D levels		Schizophrenia	Bipolar disorder type 1	Methamphetamine-induced psychotic disorder	Control group
Male	Minimum	4.0	4.9	3.0	4.0
	Median	11.8	14.5	14.5	13.7
	Maximum	36.7	71.2	43.9	22.0
Female	Minimum	3.4	3.0	3.0	4.0
	Median	6.5	6.9	4.0	6.6
	Maximum	22.9	36.9	8.2	28.7
Whole study group	Minimum	3.4	3.0	3.0	4.0
	Median	10.8	11.2	13.6	11.1
	Maximum	36.7	71.2	43.9	28.7

Serum calcium, phosphorus, PTH, and CRP levels are shown in Table 3. The average ionized calcium serum level was 8.6–10.3 mg/dl, the average phosphorus serum level was 2.5–4.5 mg/dl, the average parathormone serum level was 10–65 pg/ml or ng/L, and the normal CRP serum level was less than 5 mg/L. No significant difference was seen in serum calcium and phosphorus levels between the four study groups, the male and female participants, and the patients with and without a history of hospitalization. The PTH levels in patients with schizophrenia, bipolar disorder type 1, and methamphetamine-induced psychotic disorder were lower than in the control group (p < 0.001). However, no significant difference in the PTH level was observed between patients with and without a history of hospitalization and the male and female participants. Serum CRP levels in patients with schizophrenia, bipolar disorder type 1, and methamphetamine-induced psychotic disorder were significantly higher than in the control group (p < 0.001). However, no significant correlation was seen between CRP level and age, disease severity based on BPRS, or duration of illness.

**Table 3:** Serum calcium, phosphorus, PTH, and CRP levels

Variable		Schizophrenia	Bipolar disorder type 1	Methamphetamine-induced psychotic disorder	Control group
Calcium	Minimum	8.8	8.7	6.0	7.4
	Median	9.4	9.2	9.2	9.2
	Maximum	10.2	9.8	10.1	9.7
Phosphorus	Minimum	2.6	2.0	2.3	2.5
	Median	3.6	3.5	3.6	3.5
	Maximum	4.6	4.4	4.8	4.5
PTH	Minimum	8.10	7.10	8.40	23.70
	Median	25.08	22.35	22.00	39.85
	Maximum	69.60	79.90	64.00	99.40
CRP	Minimum	0.20	0.38	0.40	0.10
	Median	1.00	1.00	1.55	1.00
	Maximum	7.40	95.00	74.00	57.00

The chi-square test results revealed no significant distinction between first-episode psychotic patients and chronic psychotic patients in terms of calcium, phosphorus, and PTH serum levels. However, CRP serum levels were significantly higher in chronic psychotic patients compared to first-episode psychotic patients (p < 0.031). Moreover, vitamin D level was considerably lower in chronic psychotic patients compared to first-episode psychotic patients (p < 0.044) (Table 4).

**Table 4:** Comparison of Vitamin D, calcium, phosphorus, PTH, and CRP levels in first-episode psychotic patients and chronic psychotic patients

Variable		First-episode psychotic patients (N)	Chronic psychotic patients (N)	P-value
Vitamin D	≥ 20 ng/ml	3	25	0.044
	< 20 ng/ml	30	72	
Calcium	≥ 8.6 mg/dl	32	103	0.388
	< 8.6 mg/dl	1	1	
Phosphorus	≥ 2.5 mg/dl	33	102	0.520
	< 2.5 mg/dl	0	2	
PTH	≥ 10 pg/ml	32	93	0.245
	< 10 pg/ml	1	4	
CRP	< 5 mg/L	33	75	0.031
	≥ 5 mg/L	0	11	

While serum levels of vitamin D, calcium, phosphorus, and CRP did not vary significantly between first-episode psychotic patients and the healthy control group, the results of the independent t-test revealed that PTH serum levels in first-episode psychotic patients were significantly lower than those in the control group (p < 0.001) (Table 5).

**Table 5:** Comparison of Vitamin D, calcium, phosphorus, PTH, and CRP levels in first-episode psychotic patients and the control group

Variable	First-episode psychotic patients	Control group	P-value
Vitamin D	13.18 ± 12.29	11.82 ± 6.30	0.518
Calcium	9.19 ± 0.63	9.21 ± 0.40	0.829
Phosphorus	3.61 ± 0.47	3.48 ± 0.38	0.193
PTH	30.11 ± 16.11	46.20 ± 18.80	0.000
CRP	1.43 ± 0.87	2.14 ± 8.22	0.623

## Discussion

This study compared the demographic characteristics and serum levels of vitamin D, calcium, phosphorus, PTH, and CRP in three groups of patients, including schizophrenia, bipolar disorder, and methamphetamine-induced psychosis, with a control group. We found that the level of education is associated with the incidence of schizophrenia, bipolar disorder type 1, and methamphetamine-induced psychotic disorder. This finding may be attributed to the lower educational achievements of individuals with psychotic disorders in academic settings. This observation aligns with a previous study, which demonstrated significantly lower educational achievements in patients with psychotic disorders in comparison with standard control groups [32].

This study also showed that the prevalence of psychotic disorders is significantly higher in single people than in married ones. It could be argued that a supportive marital relationship can provide emotional stability and a sense of belonging, potentially tackling environmental stressors and contributing to mental well-being. Additionally, having shared responsibilities in a marriage can reduce the stress and burden caused by the challenges of life challenges. However, the impact of marriage on mental health is individualized and varies from person to person. While other studies also suggest that marriage could potentially reduce the risk of developing psychotic disorders, the findings regarding this matter remain controversial [33, 34].

This study found that most participants with psychotic disorders had a positive family history of severe psychotic disorders in their first-degree families. This can highlight the impact of genetic factors on the occurrence of these disorders. A substantial body of evidence in the literature further supports the significant role of genetic factors in the development of psychotic disorders [35–37].

Contrary to the results of many studies in this field, we did not find a statistically significant correlation between Vitamin D levels in our patients and those in the control study group. One of the possible reasons for this finding was the high prevalence of vitamin D deficiency among the general Iranian population [38]. The other possible explanation was the small sample size we considered in this study. This finding aligns with Graham et al.’s study, which compared the stored plasma vitamin D level of 20 recent-onset schizophrenia patients with 20 healthy cases and found no marked difference in the vitamin D level of patients and the healthy controls [39]. However, other studies suggest there might be a correlation between vitamin D deficiency and schizophrenia [15, 40]. While in one study, following 12 weeks of vitamin D plus probiotic supplement led to a significant improvement in total Positive and Negative Syndrome Scale (PANSS) score [41]; in the other one, there was no association between vitamin D supplementation and total PANSS score or metabolic outcome including CRP [42]. Given the conflicting results surrounding the association between vitamin D levels and mental disorders, further investigations involving larger populations are necessary to reach a more precise and conclusive understanding.

This study further demonstrated that patients with psychiatric disorders have a considerably higher CRP level than the control group. Elevated levels of CRP are linked to inflammatory processes in the body, making brain microvascular damage a significant contributing factor in developing psychotic disorders [18, 19]. According to a study, CRP can be used as a differentiative marker between manic and depressive episodes, with significantly higher levels during the manic episode [43]. In line with our results, in former similar investigations, CRP levels were significantly higher in schizophrenic patients compared to the normal population [44–46]. Furthermore, Jamilian et al. demonstrated that probiotic and selenium co-supplementation improved total PANSS score and significantly decreased CRP levels in patients with chronic schizophrenia [47]. Contrary to.

these results, Wiysokinski et al. observed no difference among patients with acute schizophrenia, unipolar depression, bipolar depression, and bipolar mania regarding CRP levels [48]. Additionally, Chetsawang et al. reported that CRP level was significantly lower in Methamphetamine-induced psychotic patients compared to the control group [49]. Given the conflicting and inconclusive nature of the existing results in the literature, further studies are required to reach a more definitive conclusion.

One intriguing aspect of this study was that some of the participants in all three patient groups were in their first episode of psychosis. Studying this population showed that vitamin D serum levels were notably lower in chronic psychotic patients compared to patients in the first episode of their psychosis. Probably, patients with a long history of psychosis do not have a proper diet to get adequate sources of vitamin D. Besides, due to social isolation and a lack of sufficient sunlight exposure, these patients had fewer sources of vitamin D than newly diagnosed patients. This comparison also highlighted that CRP serum levels were substantially higher in chronic psychotic patients than in newly diagnosed ones. High levels of CRP in patients with long-term psychosis could also be because of the long-term inflammatory processes caused by their chronic illness. A review of the available literature revealed that limited studies have compared vitamin D and CRP serum levels between newly diagnosed psychotic patients and patients in their chronic phases of psychosis. Jacomb et al. discovered significantly higher levels of CRP among chronically ill patients with schizophrenia compared to the first episode of psychosis patients.

Conversely, Bolu et al. found no significant difference in CRP levels between patients experiencing their first episode of psychosis and patients with acute exacerbation of schizophrenia [50, 51]. Also, another study indicated no significant difference between patients with the first episode of psychosis and those with chronic multiepisode psychosis [52]. Since available studies have yielded contradictory findings, further investigations are needed to understand better the difference of these markers between newly diagnosed versus chronic psychotic patients.

In our study, a comparison of the first-episode psychotic patients with the control group also found no significant differences in the average serum levels of vitamin D and CRP between patients in the first episode of psychosis and the standard control group. This outcome contradicted previous studies’ findings that assessed the relationship between vitamin D levels among psychotic disorders and a standard sample of participants [12, 53, 54]. Our results demonstrated that PTH serum levels were significantly lower in the first episode and chronic psychotic patients compared to the control group participants. Our findings challenge the current understanding in the field, providing valuable new insights. However, due to the small sample size of our study, further research with larger study populations is necessary to thoroughly investigate the serum PTH levels in newly diagnosed psychotic patients and compare them with a control population.

### Strengths and Limitations

One of the limitations of this study was the small sample size which made it challenging to conclude epidemiological results and limited study findings to this limited sample size. Another limitation was the lack of proper settings and funding for multiple testing of vitamin D and CRP levels in our patient cohort, as well as the absence of checking these markers in other mental disorders, such as depression or anxiety disorders. More studies with larger samples and multiple testing during patient admission or follow-up are needed to assess vitamin D and CRP levels in other psychotic disorders.

One of this study’s strengths was using the BPRS scale to accurately evaluate the psychopathology of mental disorders in all patient groups. In addition, BPRS scoring was done under one person’s clinical judgment, making scoring more reliable and comparable. Also, patients were between 20 and 30 years old with a disease duration of fewer than 5 years which eliminates the effect of chronic disease on vitamin D and CRP levels. Finally, by evaluating serum levels of calcium, phosphorus, and PTH in addition to vitamin D and CRP, we aimed to find a new cause for starting psychotic disorders and discovering a screening tool for differentiating chronic with new-onset psychotic disorders.

## Conclusion

The incidence of psychotic disorder is influenced by genetic factors as well as several demographic factors, including educational level and marital status. Despite previous evidence in the literature suggesting a potential association between vitamin D deficiency and psychiatric disorders, our study did not find such a correlation.

This study further demonstrated that CRP levels were highly associated with psychotic disorders. Moreover, CRP levels were considerably higher, and vitamin D levels were significantly lower in chronic psychotic patients compared to newly diagnosed psychosis, which can highlight the major effects of chronic inflammatory processes and malnutrition. This study also showed lower PTH levels in first-episode psychotic patients than in the control group. While these results are indeed promising and insightful, it is important to acknowledge the need for additional studies with larger sample sizes to conclude epidemiological results.

## Data Availability

The data and materials will be available by the corresponding author upon reasonable request.

## Abbreviations

BPRS: Brief Psychiatric Rating Scale
CRP: C-reactive protein
DALYs: Disability-adjusted life-years
DSM-IV: Diagnostic and Statistical Manual of Mental Disorders, Fourth Edition
IL-1β: Interleukin 1 beta
IL-4: Interleukin 4
IL-6: Interleukin 6
PTH: parathyroid hormone
SCID-IV: Structured Clinical Interview for DSM-IV
SPSS: Statistical Package for the Social Sciences
TGF-β: Transforming growth factor beta
